# Zebrafish: A Pharmacological Model for Learning and Memory Research

**DOI:** 10.3390/molecules27217374

**Published:** 2022-10-30

**Authors:** Jen Kit Tan, Faris Hazwan Nazar, Suzana Makpol, Seong Lin Teoh

**Affiliations:** 1 Department of Biochemistry, Faculty of Medicine, Universiti Kebangsaan Malaysia (UKM), UKM Medical Center, Kuala Lumpur 56000, Malaysia; 2 Department of Anatomy, Faculty of Medicine, Universiti Kebangsaan Malaysia (UKM), UKM Medical Center, Kuala Lumpur 56000, Malaysia

**Keywords:** zebrafish, pharmacological model, drug screening, learning and memory

## Abstract

Learning and memory are essential to organism survival and are conserved across various species, especially vertebrates. Cognitive studies involving learning and memory require using appropriate model organisms to translate relevant findings to humans. Zebrafish are becoming increasingly popular as one of the animal models for neurodegenerative diseases due to their low maintenance cost, prolific nature and amenability to genetic manipulation. More importantly, zebrafish exhibit a repertoire of neurobehaviors comparable to humans. In this review, we discuss the forms of learning and memory abilities in zebrafish and the tests used to evaluate the neurobehaviors in this species. In addition, the pharmacological studies that used zebrafish as models to screen for the effects of neuroprotective and neurotoxic compounds on cognitive performance will be summarized here. Lastly, we discuss the challenges and perspectives in establishing zebrafish as a robust model for cognitive research involving learning and memory. Zebrafish are becoming an indispensable model in learning and memory research for screening neuroprotective agents against cognitive impairment.

## 1. Introduction

Zebrafish (*Danio rerio*) are tropical fish native to southern Asia and became a vertebrate model organism in developmental biology pioneered by George Streisinger in the 1970s at the University of Oregon. Since then, zebrafish have emerged as one of the animal models in preclinical studies for understanding various physiological processes and diseases. The growing interest is attributed to the favorable features offered by this species. These include ex vivo fertilization, transparent embryos and larvae (facilitate imaging), small size (2–5 cm for adult), easy maintenance, prolific nature (more than 100 eggs produced per fish), rapid development (larvae start to swim freely at 5 days post fertilization, dpf), their accessibility for genetic manipulations such as CRISPR and high homology (70%) to the human genome [1,2,3]. These features allow the setting up of laboratory facilities and experiments at a low cost [4]. Hence, zebrafish animal models provide an unprecedented opportunity for medium- to high-throughput screening in drug discovery for numerous disease models, such as cancer, organ regeneration and neurodegenerative disorders [3,5,6].

Dementia is the progressive deterioration of cognitive functions, including learning and memory processes, beyond the usual rate of aging. Age is the major risk factor for dementia. Alzheimer disease (AD) is the most prevalent form of dementia (60–70%) that affects more than 57 million people globally [7]. The two main classes of AD are familial AD and sporadic AD. Familial AD accounts for less than 5% of AD cases. Its main hallmarks are mutations involving amyloid precursor protein, presenilin 1 and presenilin 2. Sporadic AD is associated with environmental factors and genetic susceptibilities such as the apolipoprotein E genotype [8,9,10]. Despite the intensive efforts in drug development for AD, effective interventions remain limited. Some of the main challenges are the diverse etiology and complex risk factors in AD. Furthermore, the lack of biomarkers for early diagnosis means the disease has to progress to an advanced stage until the manifestation of signs and symptoms can be identified [11]. One of the main objectives of AD research is the development of medications that can slow or improve the main symptom of AD, i.e., memory lapses.

Zebrafish show a high degree of conservation in the neuroanatomical organization and neurotransmitter signaling pathways with humans [10,11]. The dorsal, medial and lateral pallium of zebrafish correspond to the isocortex, amygdala and hippocampus in mammals, respectively. The zebrafish encephalon is divided into diencephalon (forebrain), telencephalon (midbrain) and cerebellum (hindbrain). Zebrafish also retain the main excitatory glutamatergic and inhibitory GABAergic neurotransmissions and express muscarinic cholinergic receptors [10]. Neurobehaviors related to learning and memory were first reported over two decades ago [12]. Since then, a repertoire of behaviors comparable to a human, such as locomotor activity, anxiety-like behaviors, learning, memory retention, spatial and object recognition, fear responses and social preference and interaction, has been characterized in this animal [10]. These features have opened up the chance to engage zebrafish as an alternative vertebrate model in cognitive decline-related research, especially for drug discovery.

Non-human primates and rodents are the classical animal models for behavioral studies involving cognition [13]. Non-human primates have the closest neurobehavioral profiles to humans, but these studies are the costliest among other model organisms and are subject to strict ethical considerations. Rodents have been traditionally used as genetic and pharmacological models in cognitive research, but the experimental cost limits their application in large-scale drug screening. Non-mammalian organisms, such as teleost fish, fruit flies and honeybees, are being used as a complementary model because of their low costs, which are amenable to high-throughput screening [14]. Zebrafish is the most popular non-mammalian model for cognitive research because it is a vertebrate and displays a repertoire of neurobehaviors that can be related to humans. Nonetheless, findings from zebrafish studies require further validation in mammalian models before being translated into clinical trials.

This review describes the cognitive behaviors related to learning and memory in zebrafish and the assessment tools available for these behaviors. We summarize pharmacological studies that used zebrafish models to screen for the effects of neuroprotective and neurotoxic compounds on cognitive performance. Challenges and perspectives in establishing zebrafish as a robust model to study cognitive impairment are also discussed.

## 2. Types of Learning and Memory in Zebrafish

Cognition is the mental process of acquiring knowledge and understanding through thinking, learning, memorizing and sensing. The cognitive process includes sensation, perception, motor skills, attention, memory, executive function, language and processing speed [15]. Learning and memory performance is one of the most commonly assessed cognitive domains in AD research for drug discovery. Learning is the process of acquiring new information through the formation of memory, while memory is the process of consolidating, storing and recalling the acquired information [16]. Learning and memory are not the same, but both terms are often used interchangeably, especially in animal studies, because both processes are highly interdependent and difficult to distinguish and interpret alone. In this review, learning and memory are regarded as one integrated functional domain unless specified otherwise.

Zebrafish display different learning abilities, such as associative, non-associative, social (shoaling) and motor learning. In social learning, a group of zebrafish learned faster than a single individual [17]. In motor learning, zebrafish adapted locomotor commands to execute accurate movements that relied on sensory feedback [18]. This section will only focus on associative and non-associative learning because their paradigms (especially associative learning) are widely used to assess learning and memory performance in zebrafish pharmacological models (Table 1).

### 2.1. Non-Associative Learning

Non-associative learning is a general lasting change in response strength toward a stimulus due to repeated exposure. Non-associative learning is further categorized into habituation and sensitization. Habituation is the decrease while sensitization is the increase in the animal’s response to a sensory stimulus upon continuous exposure. The changes in responses could be in the short- and long-term. Habituation has been regarded as an evolutionarily conserved behavior for optimal survival. Habituation allows the animal to disregard repeated stimuli while focusing on important stimuli such as potential predators and danger in the environment [1,19]. In the laboratory setting, prolonged exposure to a series of acoustic stimuli [19] and reduced light of the surrounding environment [26] reduced the startle response in zebrafish larvae. The larvae were habituated to the sensory stimuli and could exhibit non-associative learning as early as 5 to 7 dpf. In contrast, sensitization is not commonly reported in zebrafish studies. A previous study has used sensitization to assess drug addiction. Pisera-Fuester and coworkers showed that repeated administration of nicotine and cocaine at sub-threshold doses increased the zebrafish locomotor activity and sensitivity in a subsequent paradigm known as conditioned place preference (a contextual associative conditioning with appetitive stimulus) [20]. The sensitization procedure has potentiated the induction of addiction in the animal.

### 2.2. Associative Learning

Associative learning is the process of acquiring new information by linking two elements. This type of learning is more commonly assessed than non-associative learning in zebrafish studies. The two forms of associative learning are classical (Pavlovian) and operant (instrumental) conditioning [1,16,27]. In classical conditioning, an initially neutral stimulus is repeatedly paired with an unconditional stimulus (US) that elicits an involuntary reflex response (unconditioned response) until the neutral stimulus triggers the response upon subsequent exposure. The neutral stimulus has become the conditional stimulus (CS) that initiates the conditioned response when the animal learns to associate the CS with the US after repeated pairing.

In operant conditioning, an animal learns to correlate its voluntary behavioral responses with their consequences [28]. The term was first introduced by B.F. Skinner in 1937 to define associative learning made between a behavior and its consequence, which differed from classical conditioning [29]. The delivery of an US is determined by the behavioral response to modify (strengthen or weaken) the operant behavior. The US can be paired with a neutral stimulus until the administration of the neutral stimulus alone can trigger the conditioned response. In the end, the behavioral responses that result in favorable consequences are reinforced, whereas those that lead to undesirable outcomes are weakened.

#### 2.2.1. Classical Conditioning

Classical conditioning can be further divided into appetitive and aversive conditioning based on the nature of the US. Appetitive conditioning utilizes favorable stimuli as the US. Sison and Gerlai demonstrated that zebrafish are capable of associative learning by using food as the reward and a red card as the visual cue [30]. After the conditioning, the zebrafish spent more time in the target arm based on the position of the red card that was previously paired with the food during the training session. Although the authors classified the learning as classical conditioning, the preference was scored based on the time spent in the targeted arm. Precisely, the response should be considered as voluntary, such that the zebrafish had to perform the behavior (choose the correct arm) to receive the outcome. To our knowledge, there is hardly any paradigm that actually evaluates the classical appetitive conditioning in zebrafish, because the involuntary responses toward rewards (food or sight of conspecific) in zebrafish have not been well-characterized so far.

In aversive conditioning, the CS is paired with a fear-inducing US that leads to a fear-related conditioned response when the CS is administered alone. The nature of the CS can be contextual (location or environment) or cued (sensory like visual cues). The most commonly used aversive US in zebrafish studies is the application of electric shock (ES). Fear conditioning is the most common form of classical learning assessed in zebrafish. Valente and colleagues demonstrated that both larval and adult zebrafish could perform cued fear conditioning in a paradigm that associated a checkerboard pattern (CS) with the ES (US) [31]. After training, the fish exhibited a conditioned fear response by preferring the non ES-paired area when the visual cue was administered alone. The classical learning started as early as 3 weeks post fertilization (wpf), and the performance reached a level comparable to 1 year-old adult fish by 6 wpf. Although the authors considered this form of learning as classical conditioning, the performance index was scored based on the animal’s position and turning behavior that should be regarded as voluntary responses.

In contextual fear conditioning, a novel environment is associated with the US. Kenney and colleagues showed that contextual fear conditioning in zebrafish lasted for at least 14 days and varied in the fear extinction rates among different strains [21]. Extinction is a process in associative learning such that repeated exposure to the CS without the US will eventually reduce the conditioned response. The fear response was evaluated based on the distance traveled in the tank (locomotion activity) which can be considered as an involuntary behavior. Classical fear conditioning of TU zebrafish strain extinguished more quickly than AB and TL background strains.

Fear-related responses include freezing (immobility), erratic movements (zigzagging), bottom-dwelling, a tighter shoal, leaping (jumping) and thigmotaxis [32]. In the study by Baker and Wong, they used the alarm substance released from the fish skin when injured as an olfactory cue (US) to trigger anti-predatory (fear) responses such as freezing duration and erratic movements [22]. Interestingly, contextual fear learning and memory in zebrafish differed by the stress coping styles. Fear memory was acquired faster in zebrafish with a reactive stress coping style than in proactive fish. The authors postulated that a faster learning rate in reactive fish might facilitate memory encoding. The reactive fish are more sensitive to environmental cues by perceiving the fear stimulus as more threatening and becoming more risk-averse. This may lead to longer fear memory retention in the reactive fish as observed in the study. At the molecular level, the influence of stress coping styles on learning and memory performance could be explained by the difference in the transcriptomic state of the zebrafish brain. The reactive fish brain showed increased expression of genes related to synaptic plasticity and neurotransmission [33]. Maximino et al. reported that neurotransmitters such as epinephrine and norepinephrine in the autonomic nervous system and extracellular serotonin levels in the zebrafish brain increased after the exposure of conspecific alarm substance [34]. The study demonstrated that fear-induced responses and sympathetic activation were mediated by the serotonin transporter.

#### 2.2.2. Operant Conditioning

Operant conditioning can be classified as reinforcement or punishment, such that the paradigm increases or decreases the response to modify the strength of the response over the course of training. The reinforcement in operant conditioning can be positive (addition of reward as a reinforcer) or negative (removal of aversive stimulus as a reinforcer). Both positive and negative reinforcement paradigms are aimed at strengthening the behavioral response.

Positive reinforcement administers the appetitive US after the animal performs a behavioral response to strengthen the response. Manabe and co-workers developed an automated operant device for positive reinforcement conditioning in zebrafish. The response key was equipped with a sensor that dispenses brine shrimp eggs when it was approached by zebrafish [23]. In the paradigm, the red LED attached to the response key was first illuminated and then switched off when the fish approached the response key, followed by the dispensation of food.

Negative reinforcement removes the aversive US when the animal engages in a behavioral response. This type of conditioning can be further divided into escape learning and avoidance (active and passive). Escape learning refers to the animal engages a behavior to terminate the ongoing aversive stimulus. For example, the crossing response of rodents will be increased from a compartment with existing foot shock to the opposite compartment in a shuttle box. The active avoidance paradigm requires the presentation of a cue (visual/auditory/contextual) prior to the administration of an aversive stimulus. Active avoidance was performed to prevent the happening of the aversive stimulus. Xu and colleagues demonstrated that zebrafish increased crossing response to a compartment without ES upon presenting a light signal previously paired with the administration of ES [24]. In passive avoidance, also known as inhibitory avoidance, the conditioning suppresses an innate behavior to avoid the occurrence of an aversive stimulus. For example, zebrafish have an innate response to enter a dark environment (scototaxis) [25]. In the paradigm, the ES was applied when the fish entered the dark compartment of a tank. After the conditioning, the latency to enter the dark compartment was increased [25,35].

Similarly, punishment-based operant conditioning is divided into positive (impose punishment) and negative (remove appetitive stimulus as punishment). Both positive and negative punishment paradigms are aimed at weakening the behavioral response. For positive punishment, an aversive US is administered when a behavioral response is engaged, eventually leading to a decreased behavioral response. The difference between positive punishment, escape learning and avoidance is that the behavior response in positive punishment leads to the administration of an aversive stimulus, while the behavioral response in the latter two negative reinforcement results in the removal/prevention of an aversive stimulus. For example, in positive punishment conditioning, a rodent learns to refrain from pressing a lever that will lead to foot shock.

For negative punishment, an appetitive US is removed when the animal performs a behavioral response. For instance, this conditioning is used in dog training to correct undesired behavior by taking away the reward (food or toy). To our knowledge, both positive and negative punishments have not been engaged in pharmacological studies using zebrafish as models to assess cognitive performance.

### 2.3. Remarks on Leaning Conditionings in Zebrafish

In summary, zebrafish are capable of performing both non-associative and associative learning tasks. This allows the researcher to assess the zebrafish’s cognitive performance using various tests. From a survival perspective, operant learning allows the animals to find a safe and rewarding outcome while avoiding danger in a complex environment. Unsurprisingly, operant conditioning is pervasive in zebrafish studies because task performance could be enhanced by adding an operant component [31]. Nevertheless, not all types of operant conditioning can be assessed in zebrafish as in the rodent models due to lack of a suitable operant box that can be accessed by many laboratories, In addition, the nature (involuntary and voluntary) of some behaviors, especially for classical conditioning, in zebrafish is not clearly defined (for example, location preference, time spent). In a review by Pritchett and Brenna, the authors considered that many behavioral tasks in zebrafish are actually a hybrid of classical and operant conditioning [27]. In this review, tasks that include both involuntary and voluntary behaviors, and assess these responses from a hybrid of classical and operant conditioning are simply referred as associative learning. Precise classification of associative learning might be important when understanding the distinct neural circuits involved. Future studies are encouraged to indicate the types of associative learning if possible.

### 2.4. Memory

Memory can be classified mainly into sensory, short-term and long-term [36,37]. Sensory memory is further divided into haptic (based on sight stimuli), echoic (based on auditory stimuli) and iconic (based on touch stimuli) memory. Based on the duration of holding, memory is divided into short- and long-term. Working memory is a form of short-term memory that not only temporarily retains but manipulates the information for cognition, including learning, reasoning and language comprehension. Long-term memory can be further grouped as declarative (explicit or conscious) and non-declarative (implicit or unconscious) memory. There are two types of declarative memory, namely episodic (stores personal experience) and semantic (stores facts and conceptual knowledge) memory. The four types of non-declarative memory include procedural (recalling motor and executive skills), associative, non-associative and priming (influence of a pre-exposed/suggested stimulus on response to the next stimulus) memory.

Not all forms of memory can be evaluated in zebrafish as model organisms. Similar to rodent models, the most common types of memory assessed in zebrafish for cognitive studies are spatial, recognition and associative memory. To score the memory retention index, a time delay between the training (familiarization) and test (probe) phases is required. Spatial memory in zebrafish is assessed based on the preference of zebrafish for new space/area. The cue to a novel place could be contextual or visual (such as geometric cues of circle, triangle and square around the tank). Cognato et al. showed that spatial memory in zebrafish lasted up to 3 h after the familiarization session and diminished after 6 h of exposure [38]. Objective recognition memory in zebrafish refers to the preference of zebrafish for a new object. By using virtual objects presented via iPod, Braida et al. demonstrated that object recognition memory lasted up to 24 h post-familiarization session [39]. Interestingly, Madeira and Oliveira reported that zebrafish were capable of social recognition memory, i.e., the preference for new conspecifics, in a social discrimination paradigm [40]. The zebrafish were exposed to two conspecifics via perforated partitions such that the recognition relied on visual and olfactory cues. Zebrafish had increased exploration time toward novel conspecifics than familiar conspecifics after 24 h post-familiarization session. Associative memory in zebrafish based on passive avoidance [25] and appetitive conditioning [41] has been reported. Previous studies also explored other more complex forms of memory such as working memory [42,43] and episodic-like memory [44] in zebrafish. As these forms of memory are challenging to be defined and tested in animals, more evidence is required to confirm the presence of these forms of memory in zebrafish.

## 3. Behavioral Assessment Tests in Zebrafish

The main types of behaviors assessed in zebrafish as pharmacological models for learning and memory performance are locomotion, emotion (anxiety-like), cognition (learning, object recognition, spatial memory) and social interaction (preference, aggressiveness) [45]. (Table 2) There could be some variations when conducting a neurobehavioral paradigm in zebrafish to capture the change of innate behavioral response due to the drug treatment. For example, the habituation (familiarization) phase might be employed in a conditioning paradigm to reduce the anxiety or exploration of zebrafish toward novel environments. However, not all studies engaged a habituation phase, or the habituation duration might be varied between studies. A training phase is employed for memory acquisition and consolidation. In associative conditioning paradigms, the learning performance can be assessed throughout training if the paradigms involve multiple training sessions. For example, the effect of a drug on the behavioral response can be compared based on the difference between pre- and post-training [46]. Then, memory retention is evaluated in a probe phase which is conducted after the last training session. The time interval between the training and probe phase can be adjusted to evaluate short- and long-term memory. The probe phase is conducted without the unconditioned stimulus, while the conditioned response is recorded as an indicator of memory retention based on the difference between treated and untreated groups [47].

### 3.1. Locomotor Activity Test

The locomotor activity test (LAT) is used to evaluate the spontaneous swimming behaviors of zebrafish [48]. It is not a learning and memory test but is often conducted in line with cognitive tests because locomotor activity is involved in cognitive tests in response to stimuli. In addition, psychoactive drugs often impact locomotor activity. LAT is used to establish the baseline locomotor activity, as well as the effect of the drugs and environmental stimuli on locomotor activity, which may impact the cognitive test results later [49]. LAT is conducted by holding a single zebrafish in a trapezoid or rectangle water tank for a few minutes. The movement of zebrafish is recorded from the front (usually) or top of the tank, and the data are analyzed using tracking software. Parameters assessed in the LAT include total distance traveled, swimming speed (m/s, average or maximum), freezing time and thigmotaxis (time spent and distance traveled near the tank wall). A 3D LAT was developed to track the spatial movement of zebrafish [50].

LAT is generally performed first in a battery of behavioral tests before other more complicated paradigms. This can prevent the animals from becoming exhausted and less motivated to perform the subsequent paradigms. To our knowledge, there is no consensus on a standardized environment or environmental conditions for LAT. Most behavioral tests developed for zebrafish serve as a general guide rather than a standardized protocol. Considering that zebrafish are increasingly used as study models across different laboratories worldwide, standardization may be necessary to make the findings from different studies comparable. Recently, Maeda et al. developed a standardized method for assessing the behavioral response (including locomotor activity/response) in zebrafish larvae (6 to 7 dpf) using a light–dark locomotion test [49]. The characterized responses were proposed to be used for screening psychoactive substances. In addition, Ogi et al. suggested some recommendations/guidelines for conducting a social preference test based on their systematic review [51]. Nevertheless, we think it is challenging to follow exactly a standardized protocol such that certain modifications might be required. The adaptation could be attributed to the strain/source of zebrafish, the pharmacokinetics of drugs being tested (hence the timing to evaluate the drug efficacy concerning the paradigm) and the test apparatus and setup (water exchange frequency and temperature; the same types/setup of stimuli being not available). Therefore, it is recommended to specify these parameters as detailed as possible in the publication to improve the reproducibility of the test results. More importantly, the inclusion of positive and negative drug control groups is highly advisable.

### 3.2. Novel Tank Test

A novel tank test is, similar to the LAT setup, used to assess the anxiety-like behavior of zebrafish [48]. The tank is divided horizontally into two segments during the data analysis. Zebrafish tend to stay in the lower segment of the tank at the beginning of the test (geotaxis) and then start to explore the upper part. The reduction of time spent and distance traveled in the upper half of the tank is an indication of the anxiety-like behavior in zebrafish. Anxiogenic drugs that severely affect zebrafish exploration could impact the fish’s motivation to perform a cognitive test.

### 3.3. Inhibitory Avoidance Test

**Figure 1:**
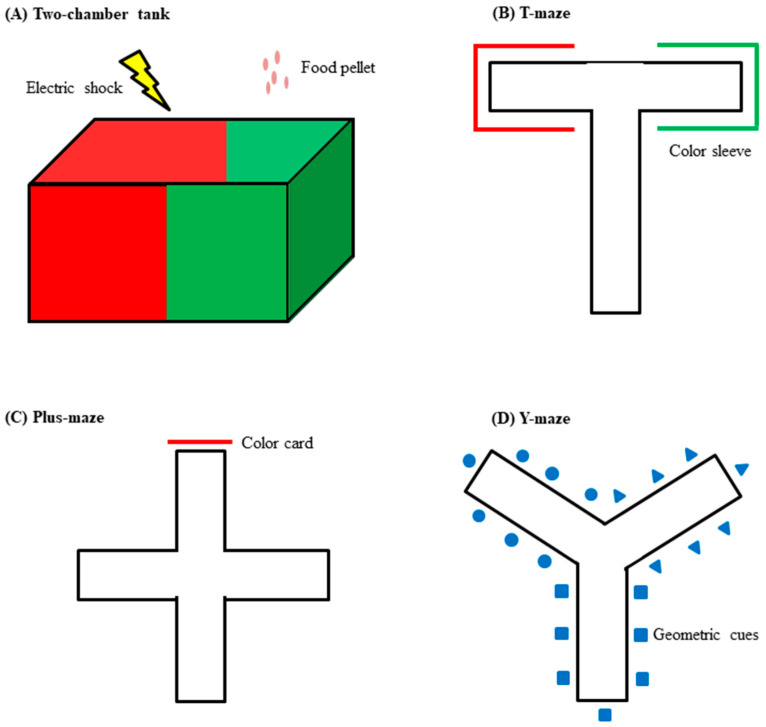
Types of tank or maze used for cognitive tests in zebrafish. (**A**) Two-chamber tank colored coded as a visual cue, for example. The unconditioned stimulus can be appetitive such as a food reward, or aversive such as an electric shock; (**B**) T-maze; (**C**) Plus-maze; (**D**) Y-maze. The arms of the mazes can be coded with different color or geometric cues.

The inhibitory avoidance test (IAT) is one of the most commonly used cognitive tests to evaluate the learning and memory functions of zebrafish. The test can be established using a two-chamber tank (Figure 1A) and a maze tank of various shapes (Figure 1B–D). In a two-chamber tank, both sides of the tank/maze are cued with color sleeves or a dark–light environment, while one side is chosen to pair the cue with an aversive stimulus such as the ES [52], dropping weight [53] or stirring [54]. In IAT, the favorable side (usually the dark compartment) is chosen for the CS–US pairing. During the training session, the zebrafish is placed in the light compartment and allowed to cross over to the dark compartment. An ES is applied to fish when it enters the dark compartment. The training session can be repeated several times until a conditioned response develops. A probe session is then conducted after 24 h (for short-term memory) of the last training trial, such that the fish is allowed to explore the compartment without the ES. The latency to enter the dark compartment during the training and probe sessions is recorded as an indication of memory retention. Other parameters such as the number of entries to and distance traveled in the dark compartment can be measured using software.

### 3.4. Appetitive Conditioning Test

Similar to IAT, a rectangle tank or maze is used to establish the CS–US pairing over repeated exposure. Chen et al. covered the left and right arms of a T-maze with green and red colored sleeves as visual cues [48]. One of the arms was used as an enriched chamber that contained brine shrimp as the appetitive stimulus. The test paradigm consisted of habituation, training and probe phases. The authors evaluated the memory performance based on the latency to enter and time spent in the targeted zone. Other appetitive stimuli can be the sight of conspecifics, favorable environment with artificial grass and stones.

### 3.5. Y-Maze Test

The Y-maze test is used to inspect the spatial memory of zebrafish based on their innate behavior that tends to explore the novel place [55]. Zebrafish were tested individually in a Y-shaped tank. Spatial memory was represented by spontaneous alternation percentage (tendency to alternate different arms in every three consecutive entries), time spent and distance traveled in the novel arm.

### 3.6. Novel Object Recognition Test

Similar to the Y-maze that is based on the fish response to novelty, a novel object recognition test is used to examine the recognition memory. In the test conducted by Capatina et al., a zebrafish was placed in a tank with two familiar objects (red cubes) and allowed to explore the tank for 10 min [55]. After an hour, one of the red cubes (familiar objects) was replaced by a green cube (novel object) and the exploration of fish was recorded. Distance less than 2.5 cm away from the objects was considered as an exploration of the objects. Recognition memory was scored by the exploratory time and the preference for the novel object.

## 4. Zebrafish Models for Pharmacological Studies on Learning and Memory

### 4.1. Neuroprotective Screening

The neurobehavioral tests for zebrafish have been reported for over two decades [12]. The utilization of zebrafish as a model to screen for compounds with a neuroprotective effect against cognitive impairment started a decade ago [53]. In recent years, zebrafish have become one of the popular animal models for screening the effects of neuroprotective drugs against various cognitive impairment models [47,53,54,55,56,57,58,59,60,61,62,63,64,65,66,67,68,69,70,71,72,73,74,75,76,77,78,79]. (Supplementary Table S1) This is attributed to the development of robust behavioral tests and well-characterized neurobehaviors and signaling pathways in zebrafish. Different zebrafish models have been developed to represent cognitive impairment in humans. These models are useful in screening for agents with neuroprotective effects that restore learning and memory performance.

Cholinergic signaling is involved in memory acquisition and consolidation. Reduction of acetylcholine (ACh) due to hydrolysis by acetylcholinesterase (AChE) could implicate the development of dementia and AD. Scopolamine is an alkaloid that acts as a nonselective muscarinic receptor antagonist. It blocks the receptors to induce cognitive impairment. Scopolamine-induced amnesia in zebrafish is a widely used model for screening drugs with neuroprotective effects [53]. Capatina et al. investigated the effects of oregano (*Origanum vulgare* spp. *hirtum* (Lamiaceae)) essential oil on the scopolamine-induced amnesia zebrafish model [55]. Scopolamine fish pre-treated with the essential oil spent longer in the novel arm of Y-maze and with the novel objects in novel object recognition test. The major components in the essential oil, such as thymol, p-cymene and γ-terpinene, could be responsible for the antioxidant activity and inhibition of AChE activity that improve cognitive performance. Devidas et al. tested the effect of 1-Hydroxy-5,5-dimethyl-5,6,7,8-tetrahydro-9,10-anthraquinone, a previously undescribed anthraquinone isolated from walnut (*Juglans regia* L.), on the scopolamine-induced cognitive deficit zebrafish model [47]. The authors found that the compound increased the fish entries and time spent in the correct arm of the T-maze following the scopolamine insult. The protective effect of the compound is likely mediated via cholinergic neurotransmission because the compound exhibited potent inhibition of AChE activity in an in vitro cell-free assay. Many other studies have utilized the scopolamine-induced cognitive impaired model in zebrafish to screen for neuroprotective agents, such as peptides from walnut protein hydrolysates [73], lithium carbonate (bipolar disorder drug) [57], sulforaphane (found in crucifereous vegetables) [72], adenosine signaling-related compounds (caffeine, ZM 241385, DPCPX, dipyridamole and EHNA) [70], flavonoids (quercetin and rutin) [71], and physostigmine (AChE inhibitor) [53].

Lim et al. used a high-cholesterol diet-induced cognitive impairment model of zebrafish to explore the effect of two lactic acid bacteria (LAB) strains: *Pediococcus acidilactici* LAB4 *and Lactobacillus plantarum* LAB12 [75]. The fish in the high-cholesterol diet groups were fed with hard-boiled egg yolk, while the LBAs were incorporated into the feed. Both LABs increased the correct response of high-cholesterol diet-fed fish for food reward in a spatial alternation task. The enhancement of spatial learning was inversely associated with amyloid precursor protein A gene expression. In addition, the LABs reduced body weight and cholesterol in serum and liver. The cholesterol-lowering properties of the LABs were related to the down-regulation of Niemann-Pick C1 Like 1 gene in the intestine that mediates cholesterol uptake, and elevation of ATP-binding cassette family transporter type A1 gene in the liver that regulates cholesterol efflux. This study uncovered the possible use of zebrafish to determine the relationship between a high-fat diet and memory impairment and the therapeutic effects of dietary supplements against the impairment. Zang et al. also demonstrated the neuroprotective effect of probiotics *Lactobacillus plantarum* ST-III using the triclosan-induced neurodegeneration zebrafish model [74]. They prepared the probiotics diet by adding the LAB into the feed. The feeding was quantitatively controlled based on the number of fish in the tank. The probiotics diet reduced distance traveled and elevated the number of transitions to the target side in T-maze for triclosan-treated fish. Cognitive improvement by the probiotics was linked to the recovery of the gut microbiome communities, lipid metabolism and intestinal mucosal immunity following triclosan exposure. These studies demonstrate the feasibility of zebrafish as a model to screen for supplements such as probiotics.

Kundap et al. developed a zebrafish model of epilepsy that induced seizure-like behaviors and cognitive dysfunction using pentylenetetrazole (PTZ) [80]. The model was used in further studies to assess the effect of embelin, a benzoquinone derived from a false black pepper (*Embelia ribes*), on acute and chronic PTZ-induced seizures and cognitive impairment. In the acute seizure model, pre-treatment of embelin (0.078–0.625 mg/kg) reduced seizures and ameliorated epilepsy-associated cognitive dysfunction induced by a single dose (170 mg/kg) of PTZ [67]. Time spent in the wrong arm and distance traveled by the PTZ-treated fish in the T-maze were decreased by embelin. Neuroprotective mechanisms of embelin may be mediated via the γ-aminobutyric acid (GABA) receptor pathway because the compound showed high affinity toward the receptor in the docking study and increased GABA level in the fish brain. In contrast, a chronic seizure model was induced by a daily dose (80 mg/kg) of PTZ for 10 days [68]. Daily pre-treatment of embelin (0.156–0.625 mg/kg) reduced the navigation time and performance errors in a three-axis maze test. Embelin increased neurotransmitters (GABA, glutamate, ACh) and showed anti-inflammatory action via the down-regulation of pro-inflammatory gene expressions (*ccl2, tlr-4, tnf-α, il-1, ifn-γ*).

Quercetin has neuroprotective properties but is limited with lower bioavailability and permeability across the blood–brain barrier. Rishitha and Muthiraman studied the efficacy of nanoparticle-formulated quercetin on PTZ-induced cognitive impairment in zebrafish [69]. Intraperitoneal injection of solid lipid nanoparticle of quercetin at 5 and 10 mg/kg prior to PTZ administration increased the time spent in the preferred chamber and decreased the number of entries to the non-preferred chamber in a two-chamber test. In another test known as the partition preference test, the time spent in and the entry to the target chamber was increased by quercetin-loaded nanoparticle pre-treatment following PTZ induction. The authors also found that quercetin-loaded nanoparticles increased time spent in the upper (preferred) segment and decreased time spent in the lower segment in a three-horizontal compartment test. The neuroprotective effect of quercetin-loaded nanoparticles is presumed to be mediated via their antioxidative properties and neurotransmitter regulation, as the drug increased the reduced glutathione and decreased the thiobarbituric acid reactive substance (a lipid peroxidation marker) and AChE activity in the fish brain.

N-Methyl-D-aspartate receptors are ionotropic glutamate receptors related to cognitive functions. MK-801, an N-Methyl-D-aspartate receptor antagonist, has been shown to induce amnesia in zebrafish [81,82,83]. On the other hand, Seibt et al. used MK-801-exposed zebrafish to determine the effect of antipsychotic drugs on neurobehaviors [66]. Antipsychotic drugs, including sulpiride and haloperidol, restored the cognitive and social interaction deficits in fish exposed to MK-801. The drugs increased the latency to enter the dark compartment with ES in the IAT and the social interaction time in the social preference test.

Alcohol consumption in higher doses can impair cognitive functions leading to amnesia or blackout. Rapid elevated ethanol levels impair memory consolidation, resulting in memory gaps for individuals during intoxication. Bertoncello et al. developed a novel ethanol-induced amnesia model in zebrafish to evaluate the effect of taurine on memory consolidation [76]. The animals were exposed to taurine for an hour, followed by 1% ethanol for another hour. Both exposures were performed by immersing the fish in beakers. The ethanol-induced deficit in memory consolidation was prevented by taurine. The ethanol-exposed fish pre-treated with taurine had longer latency to enter the punished arm with ES in an IAT. In addition, neuroinflammation triggered by chronic alcohol consumption could lead to cognitive impairment. Rajesh et al. evaluated the effect of mefenamic acid, a non-steroidal anti-inflammatory drug, on a model of chronic alcohol-induced cognitive impairment in zebrafish [54]. Mefenamic acid decreased the time spent and the number of entries into the dark compartment with water stirring in an IAT for chronic ethanol-exposed fish. AChE activity was reduced in the mefenamic acid-treated group, implying the involvement of the cholinergic anti-inflammatory pathway.

Hypoxia results in ischemic injuries to neurons and cognitive deficits. It inhibits glutamate uptake into the neurons. Urinary trypsin inhibitor (UTI) is a urinary glycoprotein that inhibits proteolytic enzymes such as hyaluronidase plasmin, α-chymotrypsin and trypsin. Kim et al. demonstrated the neuroprotective effect of UTI on a cognitively impaired zebrafish model induced by hypoxia [65]. UTI-treated fish before hypoxic condition had increased time spent in the target compartment of the T-maze. UTI reduced hypoxia-induced SA-β-galactoside and brain infarction and increased GABA_A_ receptors. Using the hypoxic zebrafish model, the same research group showed that magnesium sulfate prevented cognitive deficits and brain infarction via the up-regulation of EET4A glutamate receptor [64].

Healthy and cognitively unimpaired zebrafish are models for screening compounds or conditions with memory enhancement (nootropic) properties [46,84,85,86,87,88,89,90] (Supplementary Table S2). Yoo et al. used zebrafish to screen for the nootropic effect of UTI [90]. UTI increased the time spent, moving distance and frequency of the fish in the target compartment of the T-maze. The cognitive enhancement properties of UTI were related to the up-regulation of the protein level of EET4A glutamate receptor, and gene expressions of *c-fos* and *bdnf* in the zebrafish brain. Pusceddu et al. utilized zebrafish to evaluate the chronic effects of Mediterranean natural extracts, namely licorice (*Glycyrrhiza glabra*) root extract and rosemary extract, on cognitive functions [86]. Both extracts at lower concentrations (100 mg/L) improved the retention memory in the T-maze but induced off-target activity at higher concentrations (250 mg/L). Grossman et al. assessed the effect of piracetam, a drug widely regarded as a nootropic agent, on zebrafish [89]. The authors showed that acute piracetam exposure at 700 mg/L for 20 min inhibited swimming in a novel tank test. Chronic piracetam exposure at 200 mg/L for 7 days enhanced the cognitive performance by decreasing the latency to the target arm and increasing the duration in and the entries to the target arm. However, the chronic exposure increased total (correct and incorrect) arm entries, indicating the hypermobility effect of the drug. Echevarria et al. tested the memory-enhancing effect of methylene blue using healthy zebrafish [85]. Methylene blue showed a hormetic dose–response curve (U-shaped curve with a biphasic dose response) such that fish treated with 10 μM (high dose) methylene blue via immersion performed worse than the compound at 0.5 and 5 μM (low dose) in the T-maze. Nicotine and nicotinic agonists could enhance cognitive performance via α7 and α4β2 nicotinic acetylcholine receptors (nAChRs). Levin and Chen utilized zebrafish to determine the nicotinic involvement in learning and memory functions [88]. Acute exposure (3 min) to nicotine exhibited a biphasic effect on memory function, such that the fish performed better at a low dose (<100 mg/L) and displayed poor performance at a high dose (>100 mg/L) in an IAT. A further study from the same group found that the cognitive enhancement effect of nicotine at 100 mg/L was the most effective at 20–40 min post-administration [87]. Braida et al. leveraged the reward pathway in zebrafish to screen for newly developed α4β2 nAChR ligands as psychoactive compounds [46]. They found that the full agonists MCL-11 and MCL-28 enhanced the spatial and recognition memory of fish in an inverted U-shaped dose response. Based on the binding assay, MCL-11 was more potent than nicotine to zebrafish nAChRs. MCL-11 at 0.01 g/kg and MCL-28 at 1 g/kg administered intraperitoneally 20 min before the probe phase in the T-maze decreased the latency to reach the targeted arm. Such effects were blocked by the antagonist MCL-117, reinforcing the specific actions of these ligands on nAChRs.

### 4.2. Neurotoxicity Screening

Zebrafish are a popular model used to screen for compounds with neurotoxicity effects on learning and memory. These include metals/elements [48,52,91,92,93,94,95,96,97,98,99,100,101], environmental pollutants [102,103,104,105,106,107], stimulant drugs [108,109,110,111,112], alcohol [42,113,114] and others [81,82,83,115,116,117,118,119,120,121,122,123,124,125]. (Supplementary Table S3). Lower maintenance costs and the availability of diverse behavioral assays for zebrafish are the main reasons to conduct neurotoxicity screening using this animal model. The findings could be translated into human exposure or used to validate the mechanisms involved.

Shang et al. used zebrafish to validate the neurotoxic mechanism of occupational aluminum exposure in humans [92]. Serum aluminum in occupationally exposed workers was inversely correlated with cognitive score and the PI3K/Akt/mTOR signaling pathway. These findings were corroborated in aluminum-exposed zebrafish. Increased aluminum in the zebrafish brain was associated with impaired learning and memory performance and reduced PI3K/Akt/mTOR expression. Zebrafish housed in tank water containing aluminum trichloride for 30 days had increased latency to enter and decreased cumulative time in the targeted zone of T-maze. Similarly, Chen et al. examined the progressive effect of long-term accumulation of aluminum oxide nanoparticles on the learning and memory functions of zebrafish from embryo to adult [48]. In the study, 6 h post fertilization (hpf) embryos were exposed to the nanoparticles until 120 hpf and subjected to behavioral assays at 6 to 12 months old. Chronic embryonic exposure to the nanoparticles impaired the learning and memory performance of zebrafish in adulthood. Zebrafish exposed to the nanoparticles had a longer tendency to enter and lesser cumulative time in the targeted zone in the T-maze. The nanoparticle-induced oxidative stress might be responsible for the reduced neurotransmitters (ACh and dopamine) in the brain and the induction of neural cell death and autophagy in the telencephalon region of the zebrafish brain.

Bui Thi et al. utilized zebrafish to evaluate the effect of chronic lead exposure on neurobehaviors [101]. Low levels (50 ppb) of lead chloride (PbCl_2_) reduced the fish locomotor activity and aggressive behaviors in a 3D LAT and mirror biting test, respectively. In the mirror biting test, a mirror was located next to the tank. The aggressive behavior was scored based on biting and fast swimming, as zebrafish tend to display boldness by biting the mirror and tracing their reflection by quick movement. The PbCl_2_ induced anxiety-like behaviors in a novel tank test and altered circadian rhythm locomotor activity. Fish exposed to PbCl_2_ had reduced latency to enter the dark compartment with ES in the IAT, indicating memory loss induced by PbCl_2_ poisoning. Elevation of cortisol and reduction of serotonin and melatonin in the brain could be related to the altered neurobehaviors observed in PbCl_2_-exposed zebrafish. Sarasamma et al. developed an AD model based on zinc chloride (ZnCl_2_)-induced cognitive impairment in zebrafish [52]. ZnCl_2_ decreased the latency to enter the dark chamber with ES in an IAT for trained fish, signifying lower memory retention. Elevation of reactive oxygen species level, lipid peroxidation markers (malondialdehyde, thiobarbituric acid reactive substance and 4-hydroxynonenal) and stress hormones (catecholamine and cortisol) and reduction of melatonin and antioxidant enzyme activities (the reduced glutathione, glutathione peroxidase and superoxide dismutase) were observed in the brain following ZnCl_2_ exposure. The compound also affected neurotransmitters by increasing AChE activity, dopamine, glutamate and GABA levels, while decreasing glycine and histidine levels. More importantly, ZnCl_2_-exposed zebrafish displayed AD-like symptoms by elevating amyloid beta 42 and phosphorylated tau proteins in the brain.

Audira et al. investigated the adverse effect of chronic exposure to donepezil on cognitive functions and behaviors, using normal zebrafish as a model to represent healthy individuals without AD [125]. Long-term exposure to 2.5 ppm donepezil for 21 days reduced time spent in the punished arm in the T-maze for the trained fish, suggesting the memory enhancement effect of donepezil. The altered behaviors were associated with increased oxytocin and reduced cortisol in the brain. Although donepezil exhibited anxiolytic properties, it reduced locomotor activity and elevated aggressive behavior. An increments of reactive oxygen species and malondialdehyde in the muscle could underlie the locomotor impairment induced by donepezil. Therefore, the long-term usage of donepezil should be cautious because chronic exposure might have an adverse effect on normal individuals even though it improves memory retention.

Zebrafish larvae can be used to perform toxicity screening on learning and memory. He et al. used zebrafish larvae to investigate the effect of prenatal exposure to propofol, a widely used general anesthetic drug, on learning and memory function [118]. Propofol exposure at 6 until 48 hpf spared the spontaneous locomotor activity of the larvae but diminished the “activity decrease” in a dark-to-light cycle test. “Activity decrease” represents the response to repeated photoperiod stimulus, and the decline is considered as habituation learning exhibited by the larvae. The authors also found that propofol-induced cognitive impairment was correlated to the inhibition of axonal growth in motor neurons. Hu et al. investigated the joint effects of two pesticides (chlorpyrifos and deltamethrin) on the zebrafish larvae by co-exposing both compounds to the embryo at 2 until 144 hpf [123]. Co-exposure to the pesticides increased mortality and malformation rates, while decreased the hatching rate of the embryos. The co-exposure decreased swimming speed and adaptability to repeated stimulation in a dark-to-light cycles test, representing a deficit in habituation learning for the larvae. The co-exposure also altered metabolisms of glycerophospholipids and amino acids.

## 5. Limitations of Zebrafish as a Pharmacological Model for Learning and Memory Research

There are several concerns when using zebrafish as models for drug discovery in cognitive research. First, aqueous immersion was the major route of drug administration used in many studies, which might be least applicable to humans. Aqueous immersion was chosen probably due to the convenient setup. However, the bioavailability of the drugs is difficult to assess in zebrafish via this method. Over the years, other routes of administration, such as intraperitoneal [65,67,122], oral administration [64] and cerebroventricular injection [56,120] have been reported, which have greater translation value. The second limitation is the throughput of a behavioral test for drug screening. Zebrafish are social animals that prefer to swim in a group. Cognitive testing often requires the animal to be assessed individually, but this may incur anxiety and stress responses in the zebrafish. A longer habitation session could overcome this problem but greatly reduces the assay throughout when involving a large number of fish for drug screening. Valente et al. developed an automated platform that used an LCD screen to present the visual cues to the larvae in a group [31]. An automated setup that can run the paradigms in parallel and track them simultaneously by software have been developed. The software tracks multiple zebrafish simultaneously either as an individual in a multi-well plate or as a group in a tank. There are tools/software available for high-throughput screening of larval (mostly) and adult zebrafish behaviors, but they are mainly limited to locomotion and social behaviors [45]. These software include idTracker (trajectories, locomotor activity, social behaviors) [126], Multi-Animal Tracker (locomotor activity) [127], ToxTrac (locomotor activity) [128] and TrackId (new algorithm implemented on ToxTrac that decreased recognition error rate) [129]. Further development of the algorithm is necessary to encompass learning paradigms, for example, to automatically stop tracking individual zebrafish that have completed the conditioning in a group or parallel assay.

## 6. Future Perspectives

In this review, we have seen that zebrafish have emerged as one of the vertebrate models for cognitive research related to learning and memory functions over the past decade. The gaining popularity is supported by a repertoire of neurobehavioral displayed by the zebrafish and assessment tools available for these behaviors. Zebrafish have been proven useful as pharmacological models in drug screening for neuroprotective or neurotoxicity agents that affect learning and memory performance. Zebrafish emerged as a complementary model to rodents in preclinical studies before further testing on humans. One of the main advantages of the zebrafish model is lower maintenance costs for pharmacological screening. However, the clinically relevant routes of drug administration still require further exploration. In addition, innovative behavioral assessment apparatuses and software are needed to increase the assay throughput. By overcoming these limitations and combining the favorable features of zebrafish, this animal model will be indispensable in drug discovery research involving learning and memory.

## Figures and Tables

**Table 1:** Types of non-associative and associative learning.

Category	Subcategory	Paradigm	Further Subcategory	Behavioral Response	Example	Reference
**Non-associative learning**						
Habituation	-	Continuous exposure of stimulus	-	Decrease	Reduced zebrafish startle response to repeated sound stimulus	[19]
Sensitization	-		-	Increase	Repeated nicotine exposure increased locomotor activity and sensitivity of zebrafish	[20]
**Associative learning**						
Classical (Pavlovian)	Appetitive conditioning	Association of favorable stimulus with cue	-	Increase/Decrease (involuntary response)	Increased dog salivation upon hearing sound of bell that was previously paired with food	-
	Aversive conditioning	Association of unfavorable stimulus with cue	-	Increase/Decrease (involuntary response)	Decreased zebrafish distance traveled in tank that was previously paired with electric shock (Increased contextual fear response)	[21]
					Increased zebrafish freezing time and erratic movement in tank that was previously paired with electric shock (Increased contextual fear response)	[22]
Operant (Instrumental)	Positive reinforcement	Association of behavioral response with administration of appetitive stimulus	-	Increase	Increased zebrafish approach to a response key that was equipped with a sensor to dispense brine shrimp eggs	[23]
	Negative reinforcement	Association of behavioral response with removal of aversive stimulus	Escape learning (Engage a behavior to remove the existing aversive stimulus)	Increase	Increased rodent crossing response from a compartment with existing foot shock to the opposite compartment in a shuttle box	-
			Active avoidance (Engage a behavior to prevent the occurrence of aversive stimulus)		Increased zebrafish crossing response to a compartment without electric shock upon the presentation of light signal that was previously paired with administration of the shock	[24]
			Passive (inhibitory) avoidance (Suppress an innate behavior to prevent the occurrence of aversive stimulus)		Increased zebrafish latency to enter dark (preferred) compartment that was previously paired with electric shock	[25]
	Positive punishment	Association of behavioral response with administration of aversive stimulus	-	Decrease	Refrained from pressing a lever that will lead to foot shock for a rodent	-
	Negative punishment	Association of behavioral response with removal of appetitive stimulus	-	Decrease	Decreased the undesired behavior by taking away the reward in a dog training	-

**Table 2:** Neurobehavioral assessment tests in zebrafish.

Behavioral Test	Behavioral Domain	Procedure	Parameters Tested (unit)
Locomotor activity test	Locomotor activity	Zebrafish is allowed to swim for a certain period of time.	Total distance traveled (m) Speed (m/s) Freezing time (s) Thigmotaxis (s or m)
Novel tank test	Anxiety-like behavior And locomotor activity	Zebrafish is allowed to swim for a certain period of time. Tank is divided horizontally (virtually) into 2 segments.	Time spent and distance traveled in the upper or lower half of the tank (s) Total distance traveled (m) Speed (m/s) Freezing time (s)
Inhibitory avoidance test	Learning and memory retention	Can be consisted of habituation, training and probe phases. A form of operant conditioning with negative reinforcement.	Latency to enter the target area (s) Number of entries to the target area (%) Time spent in the target area (s) Distance traveled in the target area (m)
Appetitive conditioning test	Learning and memory retention	Can be consisted of habituation, training and probe phases. A form of associative learning with appetitive stimulus.	Latency to enter the target area (s) Number of entries to the target area (%) Time spent in the target area (s) Distance traveled in the target area (m)
Y-maze test	Spatial recognition and memory	Zebrafish is allowed to explore the maze for a certain period of time.	Spontaneous alternation (%) Time spent in novel arm (s) Distance traveled in novel arm (m)
Novel object recognition test	Object recognition and memory	Zebrafish is allowed to explore the objects for a certain period of time.	Time spent on novel object (s) Preference for novel object (%) Distance traveled from novel object (m)

## Data Availability

Not applicable.

## Supplementary Materials

The following supporting information can be downloaded at: https://www.mdpi.com/article/10.3390/molecules27217374/s1, Table S1: Cognitive impairment models of zebrafish and pharmacological studies for compounds with neuroprotective effects; Table S2: Screening for nootropic agents with memory-enhancing properties using zebrafish as model; Table S3: Neurotoxicity studies for compounds that affect learning and memory in zebrafish.
