# Supplementary material for: Zebrafish: A Pharmacological Model for Learning and Memory Research
Source: Molecules. 2022 Oct 30;27(21):7374. doi: 10.3390/molecules27217374 (PMC9657833; doi:10.3390/molecules27217374)
Supplement: Supplementary file 1 [file molecules-27-07374-s001.zip › molecules-1967201-supplementary.pdf]

Supplementary Table S1: Cognitive impairment models of zebrafish and pharmacological studies for compounds with neuroprotective effects.

CI Model	Test Drug	Zebrafish (age, strain, sex)	Grouping	Administration Dosage, Route & Duration	Behavioral Findings (Drug Effects)	Other Findings (Drug Effects)	Reference
24h light exposure	Melatonin	Adult: 1y; WT SF; both sexes	(1) Normal light cycle<br>(2) 24h light<br>(3) Melatonin<br>(4) Melatonin + 24h light	(1) melatonin: 0.232mg/L; AI for 24h<br>(2) normal (14h light/10h dark) vs 24h light exposure	(1) IAT: ↑ latency to dark compartment with ES	(1) Body: ↓ cortisol	[79]
Aβ42	Li	Adult; WT	(1) Ctl<br>(2) LiCl<br>(3) Aβ42<br>(4) LiCl + Aβ42	(1) LiCl: 100μM; AI from 1hpf to 5dpf; EW daily<br>(2) Aβ42: 10μM; CVI at 24hpf	(1) Bouncing-call avoidance test: ↑ escape response	(1) ↓ p-Tau protein	[56]
$AlCl_3$	Nec-1	Adult: 6-8mo; TU	(1) Ctl<br>(2) AlCl3<br>(3) Nec-1<br>(4) AlCl3 + Nec-1	(1) $AlCl_3$: 200μg/L; AI for 30d<br>(2) Nec-1: 15μmol/L; AI for 30d	(1) TM: ↑ latency to & accumulative time in target zone	(1) Brain: ↑ Ach; Neural cell no; mTOR & Caspase-8 mRNA<br>(2) Telencephalon: ↓ RIP1, PARP2, Bmf1 & Rab25 mRNA; RIP1 staining; restored cellular & organelles changes	[78]
Alcohol	Taurine	Adult: 4-6mo; WT SF; both sexes	(1) Ctl<br>(2) EtOH<br>(3) Taurine<br>(4) EtOH + Taurine	(1) EtOH: 1%; AI for 2min x 8d<br>(2) Taurine: 42, 150, 400mg/L; AI after EtOH for 1h, 5th-8thd of EtOH	(1) Shoaling behavior test: ↑ inter-fish & farthest neighbor distances<br>(2) IAT: ↑ latency to enter dark area with ES	-	[77]
Alcohol	Taurine	Adult: 3-5mo; WT; both sexes	(1) Ctl<br>(2) Taurine<br>(3) EtOH<br>(4) Taurine + EtOH	(1) Taurine: 42, 150, 400mg/L; after training, AI for 1h AI<br>(2) EtOH: 1%; after Taurine, AI for 1 h	(1) IAT: ↑ latency to enter dark side with ES & memory retention index	-	[76]
Alcohol	MA	Adult: 6-8mo, 4-5.5cm; WT Grey; both sexes	(1) Ctl<br>(2) EtOH<br>(3) EtOH + MA	(1) EtOH: dose escalation in 28d<br>(a) D1-12: 0.125 to 0.375%<br>(b) D13-28: 05%<br>(2) MA: 100, 200μg/L for 28d	(1) LDPT: ↑ time spent in dark compartment<br>(2) IAT: ↓ time spent in & no of entries into dark compartment with stirring	(1) Brain: ↓ AChE activity	[54]
HCD	LAB4 & LAB12	Adult: 2.5±0.5cm; male	(1) Ctl<br>(2) HCD<br>(3) HCD + LAB4<br>(4) HCD + LAB12	(1) HCD: hard-boiled egg yolk feed for 7w; 3% of total body weight<br>(2) LAB: 10^9CFU/ g hard-boiled egg yolk for 7w	(1) spatial alternation task: ↑ correct response	(1) LAB4: ↓ body weight<br>(2) LAB4 &12: ↓ serum & liver cholesterol; intestinal *npc1117* & brain *appa* mRNA<br>(2) LAB4 &12: ↑ hepatic *abca1* mRNA	[75]
Hypoxia	MgT	Adult: 4-6mo, 2.5-3.5cm, 350±50mg; WT	(1) Ctl<br>(2) Hypoxia<br>(3) MgT (4) 10 uM MgT + Hypoxia	(1) MgT: 10μM; PO prior hypoxia<br>(2) Hypoxia: hypoxic chamber for 1h & normaxic chamber for	(1) TM: ↑ time spent, distancemoved, entry frequency in target compartment	(1) ↓ cerebral infarction<br>(2) Brain: ↑ EEAT4 protein	[64]

				2h before probe test			
Hypoxia	UTI	Adult: 6-8mo, 2.5-3.5cm; WT AB & Tg (gad1b:gfp)	(1) Ctl<br>(2) UTI<br>(3) Hypoxia<br>(4) UTI + Hypoxia	(1) UTI: 10k, 50k, 100kU/kg, IP before training<br>(2) Hypoxia: hypoxic chamber for 10±0.5min & normaxic chamber for 2h before probe test	(1) TM: ↑ time spent in target compartment	(1) ↓ brain infarction, SA-β-galactosidase level<br>(2) ↑ $GABA_A$ receptors	[65]
MK-801	PH	Adult: 2.5bm	(1) Ctl<br>(2) PH<br>(3) SCOP<br>(4) PH + SCOP	(1) MK-801: 20μM; AI for 30min before test<br>(2) PH: 20μM ; AI for 1h min before test	(1) IAT: ↑ crossing time to the compartment with dropping stone	-	[58]
MK-801	Taurine	Adult: 4mo; SF; both sexes	(1) Ctl<br>(2) Taurine<br>(3) MK-801<br>(4) Taurine + MK-801	(1) Taurine: 42, 150, 400mg/L; after training, AI for 1h<br>(2) MK-801: 2mg/kg; after Taurine, AI for 1 h	(1) IAT: all doses ↑ latency to enter dark area with ES; 42 & 150mg/L ↑ memory retention index<br>(2) NTT: 400mg/L ↓ distance traveled & time spent in top area	-	[59]
MK-801	SUL, OLA & KAL	Adult: <8mo; male	(1) Ctl<br>(2) MK-801<br>(3) SUL, OLA or KAL only<br>(4) MK-801 + SUL, OLA or KAL	(1) MK-801; 5μM; AI for 15min before drugs<br>(2) Drugs: 9μM HAL, 50μM OLA or 250μM SUL; AI for 15min	(1) IAT: SUL & HAL ↑ latency to enter dark compartment with ES<br>(2) SPT: SUL & HAL ↑ social interaction time	-	[66]
PTZ	EMB	Adult; WT	(1) Ctl<br>(2) PTZ<br>(3) EMB + PTZ	(1) PTZ: 170mg/kg, IP<br>(2) EMB: 0.156, 0.312, 0.625, 1.25, 2.5, 5, 10mg/kg; IP	(1) TM: ↑ inflexion ratio; ↓ time spent in wrong arm and distance traveled<br>(2) NTT: ↑ distance traveled, time spent in upper tank & distance traveled in upper tank	(1) ↓ seizure score & latency for seizure onset<br>(2) Docking: ↑ affinity to $GABA_A$ receptor<br>(3) Brain: ↑ GABA & ACh; ↓ Glu	[67]
PTZ	EMB	Adult; WT AB (SF)	(1) Ctl<br>(2) PTZ<br>(3) EMB + PTZ	(1) PTZ: 80mg/kg, IP for 10d<br>(2) EMB: 0.156, 0.312, 0.62 mg/kg, IP before PTZ daily dose	(1) 3-axis maze test: ↑ maze navigation time; ↓ performance errors	(1) ↓ seizure score<br>(2) Brain: ↑ GABA, ACh & Glu; ↓ CCL-2, TLR-4, TNF-a, IL-1 & IFN-g mRNA	[68]
PTZ	SLN-Q	Adult: <8mo, 1-1.2g; WT; male	(1) Ctl<br>(2) PTZ<br>(3) Q + PTZ<br>(3) SLN-Q + PTZ	(1) Q: 5, 10mg/kg; 30min prior PTZ<br>(2) SLN-Q: 5, 10mg/kg; IP, 30min prior PTZ<br>(3) PTZ: 7.5mM; AI for 3-4min	(1) LDPT: ↑ time spent in light (preferred) chamber; ↓ number of entry to dark chamber<br>(2) Partition preference test: ↑ time spent in and entry to target chamber<br>(3) 3 horizontal compartment test: ↑ time spent in upper (preferred) segment and ↓ time spent in lower segment<br>(4) Others: GSH, TBARS, AChE in brain	(1) Brain: ↑GSH; ↓TBARS & AChE activity	[69]
SCOP	C1	Adult: 4-6mo	(1) Ctl<br>(2) SCOP<br>(3) C1 + SCOP<br>(4) DPZ + SCOP	(1) C1: 15, 30μM; 1 h before SCOP<br>(2) SCOP: 200μM; AI for 1 h before training	(1) TM: ↑ entries and time spent in correct arm	(1) In vitro: ↓ AChE activity<br>(2) Larvae: ↓ acrylamide-induced neurotoxicity	[47]
SCOP	CAF, ZM 241385,	Adutl: 0.3-0.5g; WT	(1) Ctl<br>(2) CAF + SCOP	(1) CAF: 10mg/kg<br>(2) ZM241385: 10μg/kg	(1) IAT: all drugs ↓ latency to enter dark compartment with ES	-	[70]

	DPCPX, DIPI, EHNA		(3) ZM241385 + SCOP<br>(4) DPCPX + SCOP<br>(5) DIPI + SCOP<br>(6) EHNA + SCOP	(4) DPCPX: 0.5mg/kg<br>(5) DIPI: 5mg/<br>(6) EHNA: 100μg/kg<br>(7) All drugs: IP, 2h before exp<br>(8) SCOP: AI for 1h before training			
SCOP	COT & 6HLN	Adult: 6-8mo; WT SF; both sexes	(1) Ctl<br>(2) SCOP<br>(3) COT + SCOP<br>(4) 6HLN + SCOP	(1) SCOP: 100μM; AI for 30min before tests<br>(2) COT or 6HLN: 1,2mg/L; AI for 3m; after SCOP	(1) NTT:<br>(a) COT & 6HLN (both doses) ↑ distance traveled & time spent in top zone<br>(b) COT & 6HLN (1mg/L) ↑ distance traveled & velocity<br>(2) YM:<br>(a) COT (1mg/L) & 6HLN (2mg/L) ↑ time in novel arm<br>(b) COT & 6HLN (1mg/L) ↑ distance traveled<br>(c) COT & 6HLN (both doses) ↑ turn angel<br>(3) NORT: COT & 6HLN (both doses) ↑ % preference for novel object	(1) Brain: COT & 6HLN (both doses): ↓ AChE activity, MDA & carbonylated protein; ↑ SOD, CAT, GPx & GSH<br>(2) Brain mRNA<br>(a) *bdnf*: COT (both doses) & 6HLN (1mg/L) ↑<br>(b) *npy*: COT (1mg/L) & 6HLN (2mg/L) ↑<br>(c) *egr1*: COT (both doses) & 6HLN (2mg/L) ↑<br>(d) *nrf2a*: COT & 6HLN (both doses) ↑	[60]
SCOP	FAB	Adult: 3-4mo, 3-4cm; WT ST; both sexes	(1) Ctl<br>(2) FAB<br>(3) SCOP<br>(4) FAB + SCOP	(1) FAB: 1, 3, 5μg/L; AI for 8d, EW at D3, 7 & 8<br>(2) SCOP: 100μM; AI for 30 min prior NTT (D7) & YM (D8)	(1) NTT: 3 & 5μg/L ↑ time spent in top; ↓ time spent in bottom<br>(2) YM: 3 & 5μg/L ↑ spontaneous alternation %	(1) Brain:<br>(a) all doses ↓ AChE activity & MDA; ↑ CAT & GPx<br>(b) 1 & 3μg/L ↑ SOD	[61]
SCOP	Li	Adult: 6mo, WT SF; both sexes	(1) Ctl<br>(2) Li_2CO_3<br>(3) SCOP<br>(4) Li_2CO_3 + SCOP	(1) Li_2CO_3: 100 mg/L; AI for 7d, EW every 3d<br>(2) SCOP: 100μM; AI for 1h before training	(1) NTT: ↓ total distance travelled, entries & time in upper zone<br>(2) YM: ↑ time spent in novel arm	(1) Brain: ↑ AChE activity	[57]
SCOP	OEO	Adult: 3-4mo, 3-4cm; SF; both sex	(1) Ctl<br>(2) SCOP<br>(3) SCOP + OEO	(1) OEO: 25, 150, 300μL/L; AI once daily for 13d, 1h prior test<br>(2) SCOP: 100μM; 30min before each test	(1) NTT: ↓ time spent in bottom zone & freezing duration, ↑ distance traveled & average velocity<br>(2) YM: ↑ time spent in novel arm, spontaneous alternation behavior & distance traveled<br>(3) NOR: ↑ exploratory time on & preference for novel object	(1) Brain: ↑ SOD, CAT, GPx & GSH; ↓ AChE activity, portein carbonyl & MDA	[55]
SCOP	PH	Adult: 2.5cm	(1) Ctl<br>(2) PH<br>(3) SCOP<br>(4) PH + SCOP	(1) PH: 20μM, AI for 1h prior SCOP<br>(2) SCOP: 200μM, AI for 1h prior training	(1) IAT: ↑ crossing time to the dark compartment with dropping weight	-	[53]
SCOP	Q & R	Adult: <8mo; both sexes	(1) Ctl<br>(2) SCOP<br>(3) Tween-20<br>(4) Tween-20 + SCOP<br>(5) Q<br>(6) Q + SCOP	(1) Q or R: 50mg/kg in 1% Tween-20 in saline; IP; 2 hr before training<br>(2) SCOP: 200μM; AI for 1h before training	(1) IAT: ↑ latencies to enter dark compartment with ES	-	[71]

			(7) R<br>(8) R + SCOP				
SCOP	Rho	Adult; WT SF; both sexes	(1) Ctl<br>(2) Rho<br>(3) SCOP<br>(4) Rho + SCOP	(1) Rho: 1, 3, 5μg/L; AI for 1h once daily for 9d<br>(2) SCOP: 100μM; AI for 30 min prior tests	(1) NTT: all doses ↑ time spent in top, distance traveled, time spent in top/bottom ratio & distance top/bottom ratio<br>(2) YM: all doses ↑ time spent in novel arm, distance traveled & turn angle<br>(3) NORT:<br>(a) all doses ↑ exploratory time on novel object<br>(b) 3 & 5μg/L ↑ % preference for novel object	(1) Brain:<br>(a) all doses ↓ AChE activity & MDA; ↑ SOD, CAT & GPx<br>(b) 3 & 5μg/L ↓ protein carbonyl	[62]
SCOP	SFN	Adult: 6-8mo, 4-5.5cm; Grey strain; both sexes	(1) Ctl<br>(2) IAT<br>(3) IAT + SFN<br>(4) IAT + SCOP<br>(5) IAT + SFN + SCOP	(1) IAT daily for 10d<br>(2) SFN: 25μM/L; AI for 1h before SCOP<br>(3) SCOP: 400μM/L; AI for 1h before probe test	(1) IAT: ↓ time spent in & no of entries into dark compartment with stirring	-	[72]
SCOP	STF	Adult: 3-4mo, 3-4cm; WT ST; both sexes	(1) Ctl<br>(2) STF<br>(3) SCOP<br>(4) STF + SCOP	(1) STF: 10, 25, 50μL/L; AI for 8d, EW at D3, 7 & 8<br>(2) SCOP: 100μM; AI for 30 min prior NTT (D7) & YM (D8)	(1) NTT:<br>(a) 50μL/L ↑ total distance<br>(b) all doses ↑ time spent & no of entries in top; ↓ latency to top<br>(c) 20 & 50μL/L ↓ time spent & no of entries to bottom<br>(2) YM: all doses ↑ no of arm entries & spontaneous alternation %	(1) Brain: ↓ AChE activity 7 MDA; ↑ SOD, CAT & GPx	[63]
SCOP	WPHs	Adult: 6mo; WT AB	(1) Ctl<br>(2) SCOP<br>(3) FY + SCOP<br>(4) SGFDAE + SCOP	(1) WPHs: 20mg/L FY or 20mg/L SGFDAE; AI for 7d, EW every 2d<br>(2) SCOP: 200μM; AI for 1h after training	(1) TM: WPHs ↑ time spent in target reservoir, latency to locate reservoir & total distance traveled	(1) Docking: WPHs ↑ binding affinity to AChE & Keap1	[73]
TCS	ST-III	Adult; WT AB	(1) Ctl<br>(2) TCS<br>(3) ST-III<br>(4) TCS + ST-III	(1) TCS: 0.002% (v/v); A for 90dI, EW every 2d<br>(2) ST-III: added into basal diet	(1) TM: ↓ distance traveled; ↑ transition no to targeted side<br>(2) Bottom dwelling test: ↑ swim velocity & activity	(1) Restored intestinal flora structure & biodiversity.<br>(2) ↓ intestinal lipid peroxidation & liver lipid accumulation<br>(3) ↓ intestinal inflammation & immune response stimulation	[74]

6HLN: 6-hydroxy-L-nicotine; AB: AB strain; ACh: acetylcholine; AChE: acetylcholinesterase; AI: aqueous immersion; AlCl3: aluminium trichloride; C1: 1-Hydroxy-5,5-dimethyl-5,6,7,8-tetrahydro-9,10-anthraquinone (compound 1); CAF: caffeine; CAT: catalase; CI model: cognitve impairment model; COT: cotinine; Ctl: control; CVI: cerebroventricular injection; DIPI: dipyridamole; DPCPX: 8-cyclopentyl-1,3-dipropylxanthine; DPZ: donepezil; EAAT: Excitatory amino-acid transporter 4 ; EHNA: erythro-9-(2-hydroxy-3-nonyl)-adenine hydrochloride; EMB: Embelin; ES: electric shock; EtOH: ethanol; EW: exchange water/medium; FAB: agathisflavone; GABA: gamma-aminobutyric acid ; GGRE: Glycyrrhiza glabra root extract ; Glu: glutamate; GPx: glutathione peroxidase; GSH: reduced glutathione; HCD: high-cholesterol diet; IAT: inhibitory avoidance test IP: intraperitoneal injection; KAL: haloperidol; LAB12: Lactobacillus 1 plantarum LAB12; LAB4: Pediococcus acidilactici LAB4 ; LDPT: light-dark preference test; Li: lithium; Li2CO3: lithium carbonate ; LiCl: lithium chloride; LPS: lipopolysaccharide; MA: mefenamic acid ; MB: methylene blue ; MB-005: 1,3-diaminopropane ; MDA: malondialdehyde; MeHg: methylmercury ; mgT: Magnesium L-threonate ; MNE: Mediterranean natural extracts ; mo: months; Nec-1: Necrostatin-1 ; NTT: novel tank test; OEO: Origanum vulgare ssp. hirtum (Lamiaceae) Essential Oil; OLA: olanzapine; PCM: piracetam; PH: physostigmine ; PM: plus-maze test; PO: oral administration; PTZ: pentylenetetrazole ; Q: quercetin; R: rutin; RE:

Rosemary extract ; Rho: rhoifolin; SCOP: scopolamine; SeMet: selenomethionine ; SF: Short-fin strain; SFN: sulforaphane; SLN-Q: solid lipid nanoparticle of quercetin ; SOD: superoxide dismutase; SPT: social preference test; ST: short-thin strain; STF:Schinus terebinthifolius essential oil; ST-II: probiotics Lactobacillus plantarum ST-III; SUL: sulpiride; TBARS: thiobarbituric acid reactive substances; TCS: triclosan; Tg: trangenic strain; TM: T-maze test; TU: Tuebingen strain; UTI: Ulinastatin; WPHs: peptides from walnut (Juglans regia) protein hydrolysates; WT: wild-type; YM: Y-maze test; ZM 241385: 4-(2-[7-amino-2-{2-furyl}{1,2,4}triazolo-{2,3-a}{1,3,5}triazin-5-yl-amino]ethyl) phenol

Supplementary Table S2: Screening for nootropic agents with memory-enhancing properties using zebrafish as model.

Test Drug	Zebrafish (age, strain, sex)	Grouping & Dosage	Administration Route & Duration	Behavioral Findings	Other Findings	Reference
MB-005	Adult; WT SF; both sexes	(1) Acute: 0, 700mg/L<br>(2) Chronic: 0, 200mg/L	(1) Acute: AI for 20 min<br>(2) Chronic: AI for 14d	Acute treatments<br>(1) IAT: ↓ incorrect entries; ↑ latency to incorrect compartment<br><br>Chronic treatment<br>(1) IAT: ↓ latency to incorrect compartment; ↑ duration in and frequency to incorrect compartment<br>(2) PM: ↑ freezing duration	-	[84]
MB	Adult: 3-6mo; WT	0, 0.1, 0.5, 5.0, 10μM	AI for 12h, immediately after last day of training; returned to home tank for 12h before probe test	(1) TM: 10μM ↓ performance (vs other doses)	-	[85]
MCL-11, MCL-28 & MCL-54	Adulr: 6-12mo, 3-4cm; WT SF; both sexes	(1) MCL-11: 0.00001-1 g/kg<br>(2) MCL-28: 0.1-1000 g/kg<br>(3) MCL-54: 0.5-20mg/kg	IP 20 min before TM & VORT first probe trial; immediately before CPP conditioning phase	(1) TM: MCL11 & MCl-28 ↓ latency to EC with an inverted U-shaped dose-response curve<br>(2) VORT: MCL11 & MCl-28 ↑ DI<br>(3) MCL-54 induced CPP	(1) Binding assay: MCL-11most potent	[46]
MNE	Adult: 6-8mo, 3-4cm, 0.4-1g; WT SF; both sexes	(1) GGRE: 0, 100, 250mg/L<br>(2) RE: 0, 100, 250mg/L	AI for 30 min	(1) TM: ↓ latency to find target arm	-	[86]
NIC	Adult	0, 50, 100, 200mg/L,	AI for 3min, delay 0 -160min prior testing	(1) IAT: 100mg/L ↑ choice accuracy best at 20-40 min post administration; nicotinic antagonist (mecamylamine) reversed the effect when given before NIC	-	[87]
NIC	-	0, 50, 100, 150, 200, 400, 800mg/L	AL for 3min	(1) IAT: low dose (<100mg/L) ↑ choice accuracy; high dose ↓choice accuracy	-	[88]
PCM	Adult: 3-5mo; WT SF, both sexes	(1) Acute: 0, 100, 400, 700mg/L<br>(2) Chronic: 0, 200mg/L	(1) Acute: AI for 20 or 90min<br>(2) Chronic: AL for 7-8d, EW daily	(1) NTT: Acute PCM 700 mg/L ↓ time spent & entry duration in top<br>(2) PM: Chronic PCM ↓latency to target arm; ↑ duration in target arm, entries to target arm, incorrect arm and total arm	-	[89]
UTI	Adult: 4-6mo, 2.5-3.5cm; WT SF	0, 10k, 50k U/kg	IP daily for 2d before training	(1) TM: UTI 50k U/kg ↑ time, distance & frequency in target compartment<br>(2) The same dose increased gene expressions of EAAT4 glutamate transporter, c-fos & BDNF	(1) Brain: UTI 50k U/kg UTI ↑ EAAT4 protein; c-fos & BDNF mRNA	[90]

AI: aqueous immersion; Ctl: control; EAAT: Excitatory amino-acid transporter 4; EW: exchange water; GGRE: Glycyrrhiza glabra root extract ; IAT: inhibitory avoidance test; IP: intraperitoneal injection; MB: methylene blue; MB-005: 1,3-diaminopropane; MNE: Mediterranean natural extracts; mo: months; NTT: novel tank test; PCM: piracetam; PM: plus-maze test; RE: Rosemary extract; SF: Short-fin strain; TM: T-maze test; UTI: Ulinastatin; WT: wild-type

Supplementary Table S3: Neurotoxicity studies for compounds that affect learning and memory in zebrafish.

Compound Category	Test Compound	Zebrafish (age, strain, sex)	Grouping & Dosage	Administration Route & Duration	Behavioral Findings	Other Findings	Reference
Alcohol	Ethanol	Embryo; WT AB	0, 20mM (0.12%)	AI from 2-9dpf; tested at mo	(1) YM: ↓ alternation	-	[42]
Alcohol	Ethanol	Adult	0, 0.1, 0.25, 1%	Set 1: CPP<br>(1)Acute: single dose, test after 5d<br>(2) Chronic:7d, test after 5d<br><br>Set 2: Classical associative cognitive test<br>(1) Chronic: 18d + 8d during test<br>(2) Withdrawal: 15d + 3d no alcohol + 8d test without alcohol	Set 1: CPP<br>(1) Acute & chronic (0.25 & 1%): ↑ time spent at less preferred place<br><br>Set 2: Cognitive test<br>(1) Chronic: (0.25 & 1%): ↓ approaching index to feeding area	-	[113]
Alcohol	Ethanol	Embryo; AB	0, 0.25, 0.5, 0.75, 1%	AI for 2h, at 16hpf; tested at 6mo	(1) PM: all doses ↓ time spent in the target zone with food reward	-	[114]
Amino acid	Methionine	Adult: WT	0, 1.5 & 3mM	(1) Short-term: AI for 1h<br>(2) Long-term: AI for 7d, EW daily	(1) LAT: long-term (3mM) ↓ latency to cross to dark compartment with ES	(1) Brain: long-term (3mM) ↑ AChE activity; long-term (both doses) ↓ AChE mRNA	[115]
Drug	DPZ	Adult: 5-6mo; WT AB	0, 1, 2.5ppm	AI for 21d, EW daily	(1TM: 2.5ppm ↓ time spent in punish chamber with ES<br>(2) NTT: 2.5ppm ↑ freezing time movement ratio; both doses ↑ time , entries to & distance traveled in top area<br>(3) SPT: 1ppm ↑ interaction time<br>(4) 2.5ppm ↓ shoaling formation	(1) Brain:<br>(a) 2.5ppm: ↑ MDA; ↓ cortisol<br>(b) 1ppm: ↓ oxytocin<br>(2) Muscle: 2.5ppm ↑ MDA & ROS	[125]
Drug	MK-801	Adult: 3-6mo; AB	0, 20μM	AI for 1h for d	(1) IAT: ↓ latency to enter deep compartment with ES	(1) Brain: ↑ p-ERK	[82]
Drug	MK-801	Adult:, 6-8mo; WT SF; both sexes	0, 20μM	AI for 30min before training, after training or before probe test	(1) PM: MK-801 administered after training or before probe test ↓ time spent in target compartment	-	[83]
Drug	MK-801	Adult: 6-8mo, WT SF, equal sexes	0, 2, 20, 100μM	AI for 30min before, during or after test	(1) NTT: 100μM before or during test ↑ clock-wise circling; 100μM during test ↑ time spent in bottle layer<br>(2) Group preference test: 100μM during test ↓ time spent near stimulus fish	-	[116]
Drug	MK-801	Adult	0, 2, 20μM	AI for 37min or 1h	(1) LAT: ↑ circling behavior; ↓ swimming activity<br>(2) TM: ↓ place preference	-	[81]
Drug-Anesthetic	Ketamine	Adult: 6mo; WT SF; both sexes	0, 2, 20, 40mg/L	AI for 20 min after training<br>(1) Acute: 1d<br>(2) Repeated: 7d, once daily	(1) IAT: Acute 40mg/L ↓ latency to enter dark area with ES<br>(2) NTT: Repeated 40mg/L ↑ circling behavior	-	[117]

Drug-Anesthetic	Propofol	Larvae: 6hpf; WT AB & NBT Tg [NBT:MAPT-GFP)zc1])	0, 1, 2, 4μg/mL	AI at 6 till 48hpf	(1) LDLAT: 4μg/mL ↓ activity response to LD cycle	(1) all doses ↓ axonal length ofmotor neurons	[118]
Drug-Antidepressant	Citalopram	Larvae & adult; WT AB	0, 0.1, 1, 10, 100μg/L	AI; 2 generations exposure:<br>(1) F0⁺ larvae: 0-80hpf<br>(2) F0⁺ adult: 0-150dpf<br>(3) F1⁺ larvae: 0-80hpf<br>(4) F1⁻ larvae: 0-80hpf without drug	(1) Swimming behavior test, F1⁺ larvae: 100μg/L ↓ no of touch response; max speed & acceleration<br>(2) Open field test, F0⁺ adult male: 1, 10, 100μg/L ↓ max velocity<br><br>(3) Stimulus response test:<br>(a) F0⁺ adult female: 1μg/L ↑ max velocity to dark stimulus<br>(b) F0⁺ adult male: ↓ max velocity to dark (0.1, 10, 100μg/L), light (100μg/L), noise (10, 100μg/L) stimulus<br><br>(4) Modified TM:<br>(a) F0⁺ adult female: 10μg/L ↑ latency to correct tunnel<br>(b) F0⁺ adult male: 100μg/L ↑ latency to correct tunnel; 10, 100μg/L ↑ time spent in reward chamber	(1) spinalmotor neurons: 100μg/L ↓ length axonal projections in F1⁺ larvae<br>(2) spinal cord: 100μg/L ↓ no of Glu⁼/Hb9⁼ neurons in F0⁺ adult male<br>(3) ventral telencephalon: 100μg/L ↓ no of TH-labeled dopaminergic neurons in F0⁺ adult male	[119]
Environmental Pollutant	ACE	Adult: 9mo; WT AB	0, 1, 10, 100mg/L	AI for 2mo, EW daily	(1) LDPT: ↑ time spent in dark chamber, distance traveled, & thigmotaxis<br>(2) NTT: ↓ time & distance traveled in top segment<br>(3) NORT: ↓ time spent & distance traveled in novel object zone<br>(4) SPT: ↑ time spent & distance traveled in zone without conspecifics<br>(5) CPP: ↓ time spent & distance traveled in preferred chamber	-	[102]
Environmental Pollutant	B[a]P	Larvae & Adult (15-18mo); WT 5D Tropical & AHR2-null (ahr2hu3335)	0, 1, 5, 10, 20, 25, 25, 30, 40μM	AI from 6-120hpf	(1) Larval photomotor response test: hyperactive swimming in WT<br>(2) AAT: ↓ time spent in non ES side	(1) LPR (120 hpf larvae): hyperactive swimming in WT, no change in mutant strain<br>(2)AAT (15-18mo adults): dec time spent on non-shock side	[103]
Environmental Pollutant	BDE-47	Adult: 5-6mo; WT AB	0, 10, 50, 100, 500μg/L	AI for D15-35, 1/3 EW twice a day	(1) TM: ↑ latency in the start zone, latency to reach reward zone, freezing bouts; ↓ latency to stay in reward zone, reward arm ratio selection, instantaneous velocity	(1) Brain: ↑ *bcl-2, c-fos, grin1b, lingo 1b* mRNA	[104]
Environmental Pollutant	BP1	Larvae & adult (6mo; male); WT AB, Tg (*vmat2:GFP*) & Tg (*elavl3:GFP*)	(1) Larvae: 0, 0.8, 1, 1.2, 1.6, 2.4μg/mL (2) Adult: 0, 1, 10, 100, 1000μg/L	(1) Larvae: AI at 4dpf for 148h<br>(2) AI for 14d; EW daily	Larvae:<br>(1) ↑ embryo mortality, deformity rate, CNS & doparminergic neurons'developmental defects; ↓ hatching rate<br>(2) LAT: ↓ distance traveled & average speed<br>(3) LD challenge test: ↓ velocity & distance moved	Larvae:<br>(1) mRNA: ↑ *mbpa & pmoc*; ↓ *gfap & bdnf*<br><br>Adult:<br>(1) TM: ↑ latency to deep-water zone; ↓ deep water area/total area duration ratio<br>(2) NTT: ↓ total distance; ↑ bottom/total duration & distance<br>(3) Brain: ↓ *bdnf*; *gfap, mbpa & pomc* (mixed result)	[105]

Environmental Pollutant	Bisphenol F	Adult: 9-11mo; WT AB; male	0, 0.001, 0.01, 0.1mg/L	AI for 28d connected to automated EW system	(1) NTT: ↑ cumulative time in immobile state; ↓ no of transition to top zone<br>(2) TM: ↑ distance traveled & cumulative time in non-target arm	(1) ↑ in blood & brain<br>(2) RNA-seq: affected various pathways in brain<br>(3) Brain: ↑ choline, 17β-estradiol & cortisol; ↓ kynurenine, pregnenolone-sulfate & dehydroepiandrosterone-sulfate, indoleamine 2,3-dioxygenase & tryptophan-2,3-dioxygenase activity	[106]
Environmental Pollutant	Butyl-paraben	Adult: 10-12mo; WT AB; male	0, 0.01, 0.1, 1mg/L	AI for 28d connected to automated EW system	(1) Photomotor response test: 1mg/L ↓ photosensitivity<br>(2) TM: 0.1 & 1mg/L ↑ distance moved & time spent in non-target arm	(1) ↑ in blood & brain<br>(2) RNA-seq: affected various pathways in brain<br>(3) Brain: ↑ cortisol & cortisone; ↓ allopregnanolone	[107]
Metal	Al	Adult: 3-4mo; both sexes	0, 50, 100, 200μg/L $AlCl_3$	4d	(1) NTT: ↓ distance traveled, no of entries to top zone; ↑ time spent in bottom zone<br>(2) TM: ↑ latency to favorable zone, time spent & no of entries to unfavorable zone<br>(3) NORT: ↓ exploration time with novel object	(1) Brain: ↓ GSH, SOD, GABA, DA, noradrenaline & serotonin; ↑ MDA & glu<br>(2) IHC: ↑ no of pyknotic neurons; ↓ Nrf2	[91]
Metal	Al	Adult: 6mo; WT TU	0, 20, 100, 500μg/L $AlCl_3$	AI for 30d	(1) TM: ↑ latency to enter & ↓ cumulative time in EC	(1) Brain: ↓ *PI3K, Akt & mTOR* mRNA, neural cells; ↑ Al	[92]
Metal	Al_2O_3-NPs	WT TU	6.25, 12.5, 25, 50, 100mg/L	AI from 6 till 120hpf; tested at 6, 9, 12mo	(1) TM: ↑ latency to enter EC & ↓ cumulative time in EC at 6 & 12mo<br>(2) LAT: ↓ speed & time spent in outer zone; ↑ freezing timemovement ratio at 6, 9 & 12mo<br>(3) NTT: ↓ speed & time spent in outer zone; ↑ freezing time ratio at 6, 9 & 12mo	(1) ↑ Al in the brain at 6 & 12mo<br>(2) Brain: ↓ ACh & DA; ↑ ROS & LDH at 6 & 12mo<br>(3) Telencephalon region: ↑ neural cell death and autophagy at 6mo	[48]
Metal	Ag	Adult; AB	0, 10, 30, 50, 100, 1000ppb $AgNO_3$	AI for 72h; EW daily	(1) NTT: 30ppb ↓ distance traveled<br>(2) Swimming performance test: 30ppb ↓ swimming endurance<br>(2) SPT: 10 ppb ↓ time spent in conspecific zone<br>(3) SRT: 10 ppb ↓ time spent in familiar zone<br>(4) AAT: 10 ppb ↓ leaner %; ↑ trial no, latency of avoidance	(1) 30 & ≥50ppb: ↑mortality rate<br>(2) 10 & 30 ppb: ↑ Ag in brain<br>(3) ↓ c-fos mRNA in medial and dorsal zones of the dorsal telencephalic area	[93]
Metal	As	Embryo (24hpf), larvae (7dpf), juvenile (30 & 90dpf) & adult (150dpf); WT	0, 50, 500ppb	AI: 4 hpf to 5mo	(1) ↓ larval spontaneous tail coiling<br>(2) Adultmotor activity: hyperactive<br>(3) Juvenile & adult NTT: ↓ time spent in top zone<br>(4) SRT: hyperactive (5) Adult IAT: ↓ latency to black compartment with ES	-	[94]
Metal	As	0.3 g; both sexes	0, 1, 10, 100μg/L	AI for 96h after training	(1) IAT: ↓ latency to enter dark compartment with ES	-	[95]
Metal	Cu	Adult; WT	0, 0.77, 1.52μM $CuSO_4$	AI for 10d, EW every 2d	(1) NTT: 1.52μM ↓ velocity at D10; ↑ freezing bouts; increased latency to enter upper zone<br>(2) TM: both doses ↑ time to complete trial; ↓ correct choice	(1) Body: ↑ Cu	[96]

Metal	Cu	Larvae: 72hpf & adult; WT TU; both sexes	0, 5, 9, 20, 60μg/L $CuCl_2$	AI for 96h, EW every 2d	Larva:<br>(1) ↓ body length; ↑ distance traveled, velocity<br>Adult:<br>(1) YM: ↓ time spent in start & other arms<br>(2) IAT: ↓ latency to enter dark compartment with ES	(1) Gills: ↓ GST & GPx	[97]
Metal	Hg	Adult: 6-8mo; LF; both sexes	0, 1, 5μg/L CH3HgCl	AI for 2, 16, 12, 24, 32h	(1) Swimming performance test: ↓ distance traveled, speed, max acceleration, full clockwise rotation numbers, alter activity/inactivity period<br>(2) MBT: ↓ distancemoved<br>(3) TM: ↓ associative learning	(1) Body: ↑ SDO & MDA	[98]
Metal	Mn	Larvae: dpf & adult: 6-8mo, 0.2-0.4g; WT TU	$MnCl_2$:<br>(1) Larva: 0, 0.1, 0.25, 0.5, 1, 1.5mM<br>(2) Adult: 0, 0.1, 0.25, 0.5, 1, 1.5mM	(1) Larvae: AI at 1hpf to 5dpf<br>(2) Adult: AI for 62h, EW daily	(1) Larval exploratory behavior test: ↓ distance traveled, absolute body turn angle,movement time, immobile episodes<br>(2) Adult exploratory behavior test: ↓ line crossing, absolute turn angle, time spent in the upper tank<br>(2) Adult IAT: ↓ latency to enter dark compartment with ES	(1) Brain: ↑ TH, DOPAC, apoptosis; ↓ TH enzyme	[99]
Metal	Pb	Adult: 5-6mo; WT AB	0, 50, 100, 1000ppb (μg/L) $PbCl_2$	AI for 24 &96h, 7, 10 14 & 30d, EW every 2d	50ppb:<br>(1) 3D LAT: ↓ speed, angular velocity, total distance traveled, increased freezing timemovement ratio at D7<br>(2) NTT: ↓ entries to top compartment, ↑ freezing timemovement ratio at D30<br>(3) MBT: ↓ speed, mirror biting time at D7 & duration in mirror side at D4<br>(4) circadian rhythm LAT: altered at D30<br>(5) IAT: ↓ latency to enter dark compartment with ES at D30	(1) Brain: 50 ppb PbCl2 ↑ SOD, cortisol & MDA; ↓ AChE activity, melatonin & serotonin	[101]
Metal	Pb	Larva & Adult; WT AB	0, 0.025, 0.05mg/L PbAc	AI at 6 to 120hpf, tested at 150dpf	(1) TM: ↓accuracy rate; ↑ time to find food reward	(1) Developmental test: Induced malformation<br>(2) Spontaneousmovement test: ↓ tail bend frequency<br>(3) larval LD stimulation test: ↑ hyperactivity	[100]
Metal	Zn	Adult: 6-7mo; WT AB	$ZnCl_2$:<br>(1) Acute: 0, 2, 4, 6, 8, 10, 12, 15mg/L (ppm)<br>(2) Chronic: 0, 0.1, 0.5, 1, 1.5mg/L	(1) Acute: AI for 24, 48, 72, 96h<br>(2) Chronic AI for 21d; EW daily	(1) 3D LAT: ↓ speed, distance traveled, time spent in top tank; ↑ angular velocity, meandering, fast latent time<br>(2) MBT: ↓ mirror biting time %<br>(3) IAT: ↓ latency to dark chamber with ES<br>(4) Circadian rhythm test: ↓ circadian rhythm	(1) Brain: ↑ MDA, TBARS, 4-HNE, catecholamine, cortisol, AChE, DA, Glu, GABA, Aβ42, p-Tau; ↓ GSH, GPx, SOD, Gly, His	[52]
Peptide	Aβ42	Adult: 6mo (0.4-0.6g) & 36mo (0.8-1.2g); WT AB; both sexes	Aβ42M, O, P: 0, 10μM	CVI:<br>(1) Young (6mo)<br>(2) Old (36mo)	(1) Open tank test (7dpi): Aβ42O ↓ distance traveled in young fish<br>(2) IAT (21dpi): Aβ42O & P ↓latency to enter dark compartment with ES in young & old fish	-	[120]
Poison-neurotoxin	BMAA	Adult	0, 16.3μg/fish	IP on D0 & 7	(1) TM: ↑ latency to enter EC	-	[121]
Poison-Herbicide	Pq	Adult: 0.4-0.6g; WT; male	0, 10, 2 mg/kg,	6xIP, every 3d	(1) LAT: ↓ distance traveled, altered locomotion<br>(2) YM: ↓ time spent in novel arm	(1) Brain: ↑ DA, DAT mRNA; ↓ DOPAC	[122]

Poison-pesticide	CPF + DM	Embryo: 2 hpf; WT SB	Co-exposure of low medium, high doses:<br>(1) CPF: 4.8, 39.06, 78.13μg/L<br>(2) DM: 0.06, 1.6, 3.19μg/L	AI from 2 to 144hpf, EW daily	(1) CPF + DM ↑mortality and malformation rate; ↓ hatching rate.<br>(2) Dark-to-light stimulation test: high dose of CPF + DM ↓ swimming speed and adaptability to repeated stimulation	(1) Metabolomics: altered metabolisms of glycerophospholipids & amino acids	[123]
Sugar	Sucrose	Adult: 6-10mo; WT SF; both sexes	0, 83.25mM	AI for 14d	(1) T-plus maze test: ↓ time in and visit frequency to target arm with food reward, total no of arm visits; ↑ time showing immobility<br>(2) Spatial learning test: ↓ time in, preference toward & time spent in target zone with stimulus fish; ↑ immobile time	(1) ↑ blood glucose	[124]
Stimulant	Ayahuasca	Adult: 2.5±0.2g; WT; both sexes	0, 0.1 or 0.5ml/L	(1) Acute: AI for 1h at D14<br>(2) Chronic: AI for 1h daily for 13d	(1) One-Trial discrimination test: ↓ DI; ↑ speed & distance traveled	-	[108]
Stimulant	CAF & CBD	Larvae & Adult; WT TU; both sexes	Set 1 (CBD dose response) CBD: 0, 0.1, 0.5, 5, 10mg/kg<br><br>Set 2 (CAF long-term pre-treatment): 20mg/kg CAF + 5mg/kg CBD<br><br>Set 3 (adenosine receptor antagonists):<br>(1) Ctl<br>(2) 6mg/L Agonist (DCPX or ZM241385) + 5mg/kg CBD	Set 1: CAF, IP, 1h before or after training<br><br>Set 2: CBD, AI at 3dpf for 4mo, EW every 3 d; CAF same as set 1<br><br>Set 3: Agonist, AI for 1h; CBD same as set2	Set 1: CDB<br>(1) NTT: 0.5mg/kg ↑ time on upper zone (inverted U-shaped dose response curve)<br>(2a) IAT (CBD before training): ↓ latency to cross dark compartment with ES<br>(2b) IAT (CBD after training): 0.1, 5, 10mg/kg ↓ latency to cross dark compartment with ES; 0.5mg/kg ↑ latency to cross dark compartment with ES<br>(U-shaped dose response curve)<br><br>Set 2: CAF pre-treatment<br>(1) IAT (CBD after training): CAF ↑ latency to cross dark compartment with ES<br>for CBD pre-treated group given after training<br><br>Set 3: Agonist:<br>(1) IAT (CBD after training): ZM ↑ latency to cross dark compartment with ES	-	[109]
Stimulant	MPH + CAF	Adult: 3-6mo; WT; both sexes	Co-exposure: MPH: 80mg/L + CAF: 150mg/L	AI for 30min	(1) NTT: MPH + CAF ↓ traveled distance, crossing between zones<br>(2) SPT: MPH + CAF ↓ time in conspecific segment<br>(3) YM: MPH + CAF ↓ time in new arm	(1) Body: MPH + CAF ↑ protein carbonylation, Complexes II-III & IV activities	[110]
Stimulant	MPH	Larvae: 6dpf) AB	0, 12.5, 25, 50mg/L	AI from 0 to 5 dpf	(1) NTT: ↓ time spent in bottom 3rd of tank<br>(2) IAT: ↓ correct choice	(1) Lavae: ↑ norepinepherine, DA, serotonin	[111]
Stimulant	THC	1y age, 3-4mo; both sexes	0.1, 0.3, 3μM	AI for 1 h before test	(1) Open field maze: ↓ spatial memory	(1) Telencephalon: ↑ p-Erk	[112]

4-HNE: 4-hydroxynonenal; AAT: Active avoidance test; AB: AB strain; ACE: acesulfame; ACh: acetylcholine; AChE: acetylcholinesterase; Ag: silver; AgNO3: silver nitrate; Al2O3-NPs: Al2O3 nanoparticles; AlCl3: aluminium chloride; As: arsenic ; Aβ42: Amyloid-β42; Aβ42M: monomeric Aβ42; Aβ42O: oligomeric Aβ42;Aβ42P: protofibrillar Aβ42; B[a]P: Benzo[a]pyrene ; BDE-47: 2,2′,4,4′-tetrabromodiphenyl ether;BMAA: β-N-

methylamino-L-alanine; BP1: benzophenone 1; CAF: caffeine; CBD: cannabidiol; CH3HgCl: methylmercury (II) chloride; CNS: cental nerveous system; CPF: chlorpyrifos; CPP: conditional place preference test; Cu: copper; CuCl2: copper chloride; CuSO4: copper sulphate; CVI: cerebroventricular injection; DA: dopamine; DI: discrimination index ; DM: deltamethrin; DOPAC: 3,4-dihydroxyphenylacetic acid ; dpf: day post fertilization; dpi: days post injection; DPZ: donepezil; EC: enriched chamber ; ES: electric shock; EW: exchange water/medium; GABA: gamma-aminobutyric acid ; Glu: glutamate; Gly: glycine; GPx: gluthathione peroxidase; GSH: reduced glutathione; GST: glutathione S-transferase; HFD: high-fat diet; His: histidine; hpf: hours post fertilization; IHC: immunohistochemistry; LF: long-fin strain; LAT: locomotor activity test; LD: light-dark; LDLAT: light-dark locomotor activity test; MDA: malondialaldehyde; MBT: mirror biting test; MCL-11: $\alpha 4\beta 2$ nicotinic acetylcholine receptors (nAChRs) full agonist; MCL-117: nAChRs antagonist; MCL-28: nAChRs full agonist; MCL-54: nAChRs partial agonist; MnCl2:; Manganese(II) chloride; mo: month; MPH: Methylphenidate; NBT Tg: NBT transgenic strain; NIC: Nicotine; NORT: novel object exploration test; PbAc: Lead acetate; PbCl2: Lead Chloride; PM: plus-maze test; Pq: paraquat; ROS: reactive oxygen species; SB: SB strain; SF: short-fin strain; SOD: superoxide dismutase; SPT: social preference test ; SRT: startle response test ; TBARS: thiobarbituric acid reactive substances; Tg: trangenic strain; TH: thyrosine hydrolase; THC: Δ-9-tetrahydrocannabinol; TU: Tuebingen strain; VORT: virtual object recognition test; WT: wild-type; ZnCl2: Zinc chloride
